# Printable graphene BioFETs for DNA quantification in Lab-on-PCB microsystems

**DOI:** 10.1038/s41598-021-89367-1

**Published:** 2021-05-10

**Authors:** Sotirios Papamatthaiou, Pedro Estrela, Despina Moschou

**Affiliations:** Centre for Biosensors, Bioelectronics and Biodevices (C3Bio) and Department of Electronic & Electrical Engineering, University of Bath, Bath, BA2 7AY UK

**Keywords:** Biotechnology, Medical research, Engineering, Materials science

## Abstract

Lab-on-Chip is a technology that aims to transform the Point-of-Care (PoC) diagnostics field; nonetheless a commercial production compatible technology is yet to be established. Lab-on-Printed Circuit Board (Lab-on-PCB) is currently considered as a promising candidate technology for cost-aware but simultaneously high specification applications, requiring multi-component microsystem implementations, due to its inherent compatibility with electronics and the long-standing industrial manufacturing basis. In this work, we demonstrate the first electrolyte gated field-effect transistor (FET) DNA biosensor implemented on commercially fabricated PCB in a planar layout. Graphene ink was drop-casted to form the transistor channel and PNA probes were immobilized on the graphene channel, enabling label-free DNA detection. It is shown that the sensor can selectively detect the complementary DNA sequence, following a fully inkjet-printing compatible manufacturing process. The results demonstrate the potential for the effortless integration of FET sensors into Lab-on-PCB diagnostic platforms, paving the way for even higher sensitivity quantification than the current Lab-on-PCB state-of-the-art of passive electrode electrochemical sensing. The substitution of such biosensors with our presented FET structures, promises further reduction of the time-to-result in microsystems combining sequential DNA amplification and detection modules to few minutes, since much fewer amplification cycles are required even for low-abundance nucleic acid targets.

## Introduction

Electrochemical biosensors are considered to be at the forefront of the Point-of-Care (POC) testing due to their portability, fast response, high specificity and low cost^1^. These characteristics along with the miniaturization capability have rendered them critical elements in Lab-on-Chip (LoC) platforms. Although impressive development has been observed in the LoC field regarding their performance characteristics^2,3^ there are still certain challenges to overcome in order to achieve widespread adoption in real-life applications, principally in the remit of economy-of-scale manufacturing of such devices.

A considerable impediment in the widespread use of LoC is the use of materials not ideally fitted for the mass production of high-performance devices. Several materials have been explored as candidates for the adoption of a standardized LoC technology among the research community and the industry; nevertheless none has managed to emerge as the definitive solution to the commercial upscaling bottleneck of LoC^4^. More specifically, silicon, glass, PDMS and paper are some of the most used materials in laboratories and have exhibited satisfactory results so far. However, silicon is too expensive for mass production when cm-scale LoC devices are needed, despite the advantage of a well-established manufacturing infrastructure. Glass is transparent (convenient for optical microfluidic testing) and biocompatible, but at the same time an expensive material lagging in electronics integration. PDMS is cheap, transparent, biocompatible, flexible and versatile but similarly to glass, it lacks in electronics integration thus the cost becomes unviable for advanced quantification applications. Paper is a fairly novel material for LoC having exhibited moderate quantification sensitivity with more research required to unlock its full potential, especially in terms of microfluidic integration^4^.

Lab-on-Printed Circuit Board technology (Lab-on-PCB) has been developed for addressing the aforementioned issues, as it promises a seamless and holistic integration of electronics, sensing, fluidic handling and packaging via the already mature and well-established PCB industry^4^. PCB manufacturing is a mature and well-established industry for over 70 years. It has massively contributed to the evolution of the consumer electronics by reducing the size and the cost of the circuitry. Contemporary PCB infrastructure is capable of comparable manufacturing precision and quality to the micrometer-scale semiconductor industry. This technology claims the potential to apply the benefits it introduced to the electronics industry to the LoC field, thus promising a similar impact regarding the broadening of consumer access to bioelectronics. The effortless electronics integration that is offered by Lab-on-PCB eliminates the need for deposition methods that require expensive clean-room facilities. Indeed, this applies not only to the electrical tracks and sensing electrodes but also to the uncomplicated customization of the device with electronic components that are required, in many cases, for sensitivity and reliability improvement. Such components include micro-heaters, amplifiers, filters, optoelectronics and control circuitry. The microfluidic integration is achievable with standard PCB industry equipment and practices (or newly developed PCB compatible), aiming to produce devices ready to be used directly out of the factory. Interestingly, the usual dimensions of the microfluidic features incorporated in the bioelectronic devices are in the range of 50 μm–100 mm^4^. This characteristic perfectly matches the standard PCB machinery capabilities making redundant the highly precise and complex Si technology offering nm-scale features. In addition to this, the unification of the electronics and microfluidics manufacturing in the PCB industry mandates the same unifying practice in the design phase. Thus, the adoption of the PCB industry standard CAD software to design the microfluidic structures of the Lab-on-PCB platform is a very recent ambition^5^. Merging the electronics design with the microfluidics design in a single CAD platform will bring easier communication with the factory and unhindered implementation of the design to the manufacturing phase. Another convenient asset of the PCB industry is the fabrication of flexible printed boards, equally useful in biosensing applications^6^. Finally, environmental concerns about the disposability of Lab-on-PCBs are alleviated by the already established recycling facilities and standardized processes of the PCB industry.

Several LoC components have been showcased on PCBs^4,6^ (spanning from micropumps and microvalves to μPCR modules), including the state-of-the-art in genetic analysis, comprising seamlessly integrated rapid (10–15 min) DNA amplification and on-chip electronic detection of the amplicons^7^. However, biological field-effect transistors (BioFETs) have yet to be realized, despite their potential for even higher sensitivity and much more rapid quantification, with sensing devices limited mainly to 2–3 electrodes electrochemical biosensors^4,6^. For diagnostic applications that demand high sensitivity (e.g. cell-free nucleic acid cancer biomarkers), more sophisticated sensors are required. The BioFET is a label-free electrochemical biosensor that has been studied as a sensitive and low-cost portable proposal for a variety of biological agents’ detection^7–12^. The electrolyte gated FET is one of several FET sensor structures in which electrochemical gating is achieved through a reference electrode immersed into the solution^13–16^.

In parallel, several channel materials have been explored in BioFETs, with graphene gaining significant attention recently. Graphene, a sheet of sp_2_-bonded carbon atoms arranged into a honeycomb structure^15,17^ has been exploited as the sensing element in BioFETs owing to its unique electronic and chemical properties^13^. Graphene BioFETs display improved performance (i.e. increased transconductance) when operated in electrolyte gated mode without a solid-state gate dielectric^15,18–20^ owing to the few angstroms thick electric double layer (EDL)^18,20^ formed between the electrolyte-graphene interface. It has been found that the electrochemical gate is over two orders of magnitude more efficient than the conventional back gate with hundreds of nm thickness of SiO_2_^15,21^. High transconductance is a desirable feature for a BioFET as a tiny change in the gate bias (induced by charged analytes) will have a significant change in the channel current. Additionally, graphene is a popular choice for the active material in electrolyte gated FETs as its unique properties (e.g. direct doping from the adsorbed analytes) are better exploited by this “open” structure^22^ while being suitable for direct chemical and biological functionalization^15,22^. In terms of manufacturability, commercially available dispersions enable its effortless drop-casting or inkjet-printing deposition, thus rendering it highly compatible with PCB manufacturing processes; it is worth noting that inkjet-printed electronics processing has been converging with conventional electronics manufacturing in the last 5 years.

In this work, we demonstrate the first electrolyte gated graphene FETs (EGGFETs) on commercially manufactured PCBs. It is the first PCB based FET, utilizing the core board as the substrate and the PCB electrodes as the transistor drain, source and gate pads. Here, it has to be highlighted that the conventional, bulky, wire reference electrode is replaced by an in-plane PCB electrode for optimum miniaturization and integration. This layout is not widely explored in the literature^18,23^ where the vast majority of works make use of an external reference electrode^19,20,24,25^. The electrical behavior of the devices is investigated and a preliminary biosensing assessment is explored towards DNA detection. The DNA sequences that have been selected are oncogenic mutations of PIK3CA, one of the most commonly mutated oncogenes in human cancer^26,27^.

## Experimental

**Figure 1:**
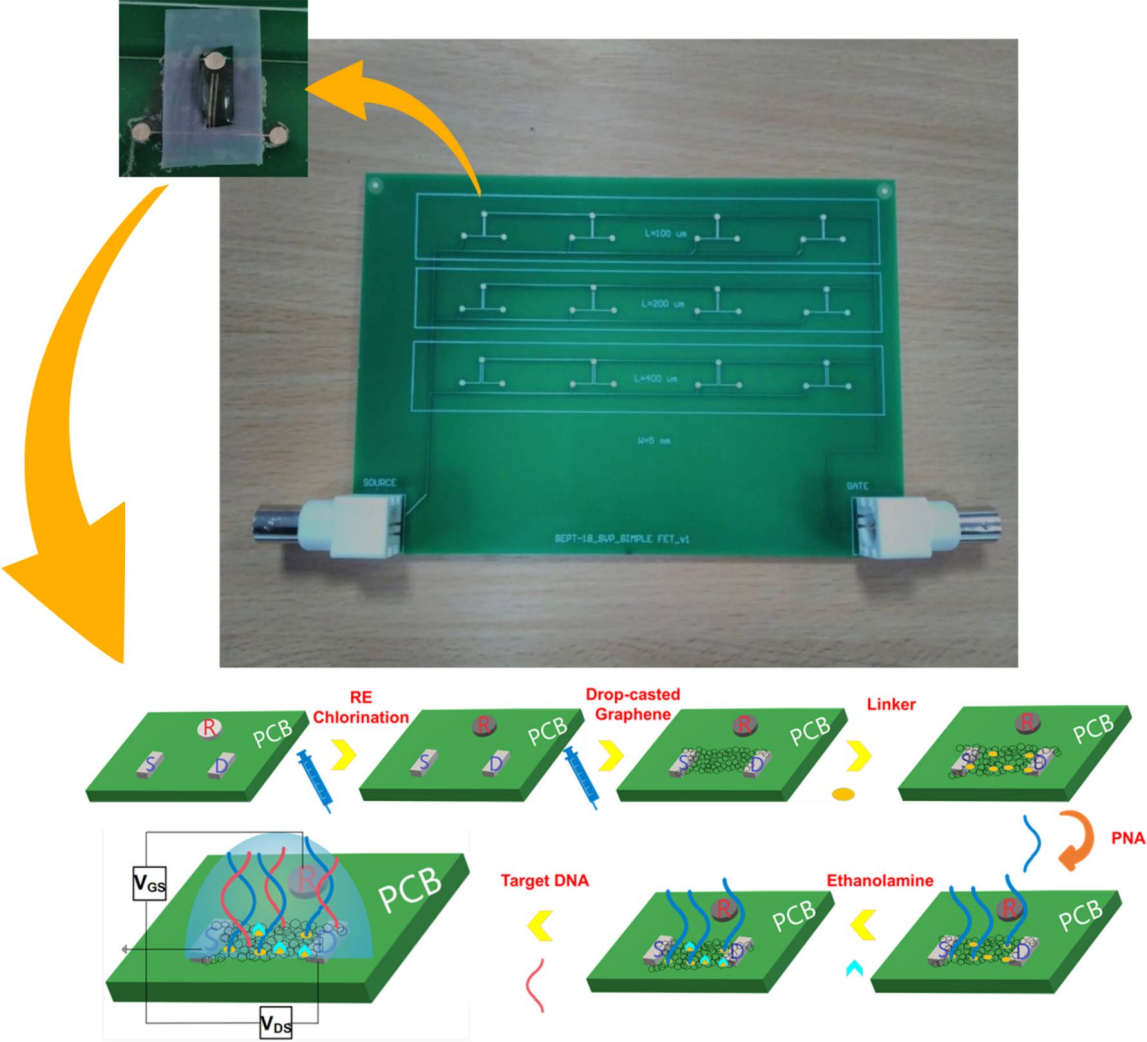
Top: Photograph of the printed circuit board (PCB) designed in Altium for evaluation of graphene as the channel material of electrolyte-gated FET. In magnification, one of the devices where the drain, source, gate electrodes are visible along with the drop-casted graphene channel. Self-adhesive Teflon tape was attached to confine the electrolyte droplet. Bottom: Schematic of assay steps for PNA-DNA hybridization detection employing an electrolyte-gated graphene field-effect transistor.

The prototype PCB platform for the EGGFET-assisted biosensing applications was designed in Altium, implementing the drain, source and reference electrodes in Ag-plated electrode format and was manufactured with commercially available technologies by Newbury Electronics Ltd, UK. Figure 1 shows the PCB with 12 devices in total. The source, drain and reference electrodes were placed in the same plane and the reference electrode was electrically insulated from the transistor channel in the absence of electrolyte droplet. The length (L) of the drain-to-source channel was 0.13 mm and the width (W) was 5 mm. The chips were briefly cleaned by rinsing them with acetone, isopropanol and distilled water. Chlorination of the silver PCB reference electrodes was achieved by drop-casting 10 μL of sodium hypochlorite solution (6–14% active chlorine) while the chip was heated at 80 °C on a hotplate until the solvent evaporated. After this stage, a change in color was observed from silver to dark grey as Figure S1 (Supplementary Information) depicts, visually indicating surface chlorination^28,29^. Similar PCB pseudo-reference electrodes from Newbury Electronics have been thoroughly evaluated previously regarding their electrical stability, proving themselves as reliable components for Lab-on-PCB platforms^28^.

Graphene ink in water with N = 13 mean number of monolayers and L = 990 nm ± 130 nm length of the flakes at 1 g/L concentration was purchased from Advanced Material Development, UK. A short sonication was performed before the drop-casting. Two successive drop-casts of 5 μL each of the graphene ink were applied between the drain and the source electrodes to form the channel while the chip was heated at 80 °C on a hotplate until the solvent evaporated.

The amine-modified PNA probe molecules were synthesized by Eurogentec, Belgium with the sequences: N-TTT CAG CCA CCA TGA CGT GCA-C. The sequences of the target complementary DNA (Sigma Aldrich) employed for hybridization were 5′-TGC ACG TCA TGG TGG CTG-3′ and the non-complementary control sequences were 5′-CAT GTA CTG CAA CAA TCA-3′. The PNA oligomers were diluted to 100 μM in distilled water and were kept below − 18 °C. DNAs were diluted to 10 μM in TE buffer (10 mM Tris–HCl, 1 mM disodium EDTA) and were kept at − 50 °C. The working principle of the EGGFET DNA sensor is illustrated in Fig. 1. In order to immobilize the PNA on the graphene, 5 mM of 1-pyrenebutanoic acid succinimidyl ester (PASE) (Sigma Aldrich) in dimethylsulfoxide (DMSO-for biological applications, Sigma Aldrich) was drop-casted on the channel to act as the linker molecule and was incubated for 1 h followed by being rinsed with 0.1 × Phosphate Buffered Saline (PBS, Sigma Aldrich). PASE binds to graphene via non-covalent π–π stacking interactions between its pyrene group and the graphene surface^30–33^. The composition of 0.1× PBS was 1 mM phosphate buffer, 0.27 mM KCl and 13.7 mM NaCl. The PASE modified graphene was treated with 10 μM PNA in 0.1× PBS for 2 h followed by being rinsed with 0.1× PBS to remove any non-immobilized probes. The N-terminus of the PNA is immobilized on the amine-reactive succinimide group of the PASE^30–33^. The neutralization of non-specific binding events was achieved by drop-casting 1 mM ethanolamine solution (Sigma Aldrich, in 0.1× PBS) for 0.5 h and washed with 0.1× PBS. The complementary PNA-DNA hybridizations were performed by dropping an appropriate DNA concentration in 0.1× PBS for 0.5 h and the non-hybridized sequences were removed by washing the channel with 0.1× PBS. The same was repeated for the control sequences. All the incubations were performed at room-temperature.

In order to get reliable and repeatable electrical measurements of the EGGFET devices, self-adhesive Teflon tape was attached around the channel to accommodate the electrolyte (0.1× PBS) in a confined space. Electrical measurements were performed inside a Faraday cage in ambient environment. The operation of the EGGFETs was controlled with a semiconductor device parameter analyzer (Keysight B1500A) using a built-in interface. The devices incorporated common reference and source electrodes while the selection of the device to be measured was performed by the corresponding drain pad. Regarding the extraction of the transfer curves, the drain to source voltage (V_ds_) was fixed to 100 mV and the maximum absolute value of the gate to source voltage (V_gs_) was always kept below 1 V. This was done to avoid any side-effects from voltage-induced chemical reactions. The V_gs_ scan rate was 50 mV/s unless otherwise stated.

## Results and discussion

### Electrical characterization of electrolyte gated graphene FETs

**Figure 2:**
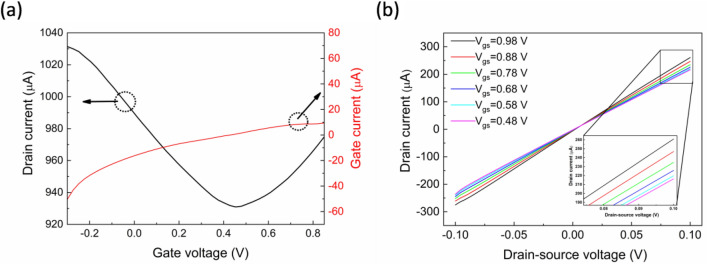
(**a**) Transfer and leakage current curves (V_ds_ = 0.1 V) and (**b**) output curves of the graphene electrolyte-gated transistor. The gate voltage is varied from 0.48 to 0.98 V with an interval of 0.1 V. Magnification in inset.

To investigate the electrical properties of our EGGFETs, the transfer characteristics were firstly extracted. The typical amphipolic graphene behavior is confirmed in Fig. 2a in a narrow V_gs_ sweep range with asymmetric n-type and p-type branches while p-type semiconductor behavior is observed as indicated by the positive magnitude of the Dirac point. The asymmetrical branches and this p-type doping are consistent with previous reports and can be attributed to the environmental exposure influence. More specifically, the adsorption of charged impurities may lead to different scattering strengths for holes and electrons, expressed by the asymmetrical nature of the transfer curves^34–36^. Also, graphene is known to be susceptible to p-type doping from water and oxygen molecule adsorption in ambient environment^30,32,34,37,38^. As it is expected for these devices, the leakage current (I_gs_) between the gate and the source is not negligible as the drain and source electrodes are not passivated to prevent contact with the electrolyte through the discontinuation of the graphene layer covering them. Nevertheless, Fig. 2a shows I_gs_ being less than 4.7% of I_ds_ at the − 0.25 V to 0 V range of V_gs_ while two orders of magnitude difference is exhibited at positive values of V_gs_. These values compare well^19^ and favorably^18^ with other EGGFETs with non-passivated electrodes in the literature. The minimal influence of the V_gs_ scan rate to the V_Dirac_ and the linear left shift of the V_Dirac_ with increasing concentration of the PBS buffer solution are also shown in Figure S2 (Supplementary Information) and are in good agreement with previously reported results^34^.

Proceeding to the output curves, Fig. 2b shows the decrease of I_ds_ with a reduction of the gate voltage from 0.98 to 0.48 V. This designates the gate influence to the channel current and reveals again the p-type doped graphene. The linear behavior of the curves indicates the lack of noticeable Schottky barriers or charge traps at the electrode–graphene interface.

**Figure 3:**
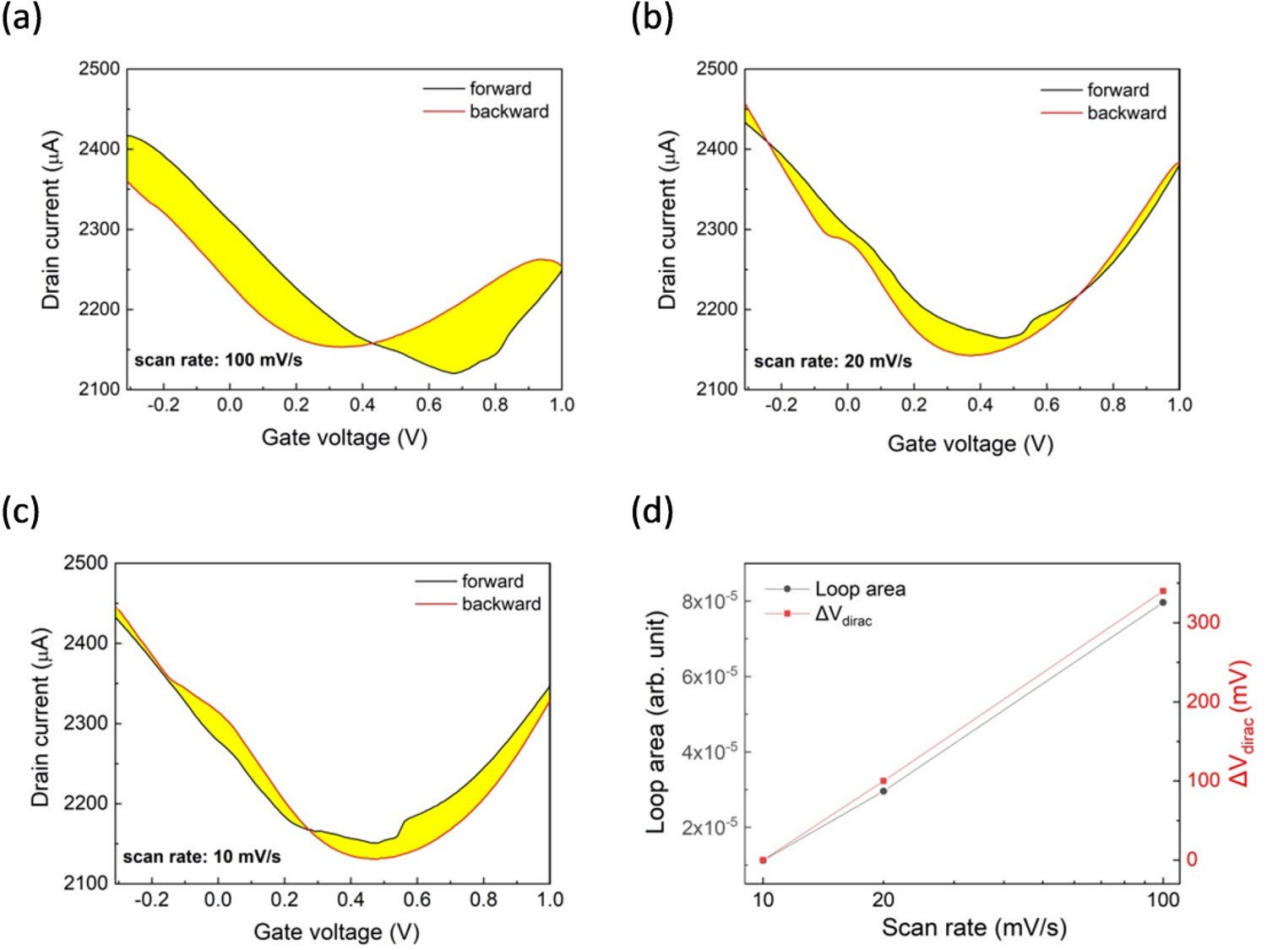
(**a**)–(**c**) Hysteresis curves of the graphene electrolyte-gated transistor for various scan rates of the sweeping gate voltage (Ag/AgCl PCB reference electrode, 0.1× PBS), (**d**) Loop area enclosed between two transfer curves by sweeping forward and backward the gate voltage and respective V_Dirac_ change as a function of the scan rate.

After having established the field-effect channel current modulation, the hysteresis characteristics were investigated. Hysteresis is a common characteristic of transistors and consequently this applies to their applications, including biosensors^39^. It is worth to study the hysteresis of an electrolyte gated transistor as hysteresis can introduce uncertainty in measuring the doping level of graphene, therefore degrading the device sensitivity and accuracy towards the detection of the analyte^39–43^. To this end, forward (starting from negative V_gs_ values) and backward (starting from positive V_gs_ values) transfer curves were performed for three different V_gs_ scan rates. For the sake of comparison, all of the investigated devices exhibited reproducible hysteresis in I_ds_ versus V_gs_ characteristics. Figure 3a,b show that the two curves tend to converge by decreasing the scan rate from 100 to 20 mV/s as also revealed by the shrunken enclosed loop area and the V_Dirac_ shift (Fig. 3d). Further decrease of the enclosed loop area is exhibited at 10 mV/s (Fig. 3c) where a left-shift hysteresis has changed to a right-shift hysteresis.

The hysteresis reduction by the scan rate decrease and the inversion between the hysteresis curves for the lowest tested scan rate indicates the co-existence of two competing mechanisms; the electrical double layer capacitance effect and the charge trapping effect. The finding of this hysteresis behavior of our drop-casted graphene on PCB devices are in agreement with Wang et al.^41^ for electrolyte gated FETs with CVD graphene transferred on SiO_2_/Si substrate, suggesting that similar mechanisms apply. Their proposed underlying mechanism for the negative hysteresis (occurred at faster V_gs_ scan rates) is the electrical double layer capacitance effect caused by the delay in the movement of ions accumulated at the graphene/electrolyte interface during the second sweep due to the “remembering” effect from the first sweep. The relaxation time of the gate potential distribution in the solution is sensitive to sweeping rate and thus slower scan rate leads to reduced hysteresis. However, the slow scan rate reveals a second hysteretic mechanism which originates from the charge trapping effect. This is the modulation of the trapped charges density by the gate voltage, which accumulate in the pre-existing defects between graphene and the substrate^41^. In this case, the hysteresis shift is positive (i.e. right shift of the second sweep curve) as negative charges are accumulated in charge traps below graphene during the first voltage sweep in the positive regime, so graphene is even more p-doped and subsequently the V_Dirac_ of the hysteresis curve is more positive.

### Evaluation of biosensing application

**Figure 4:**
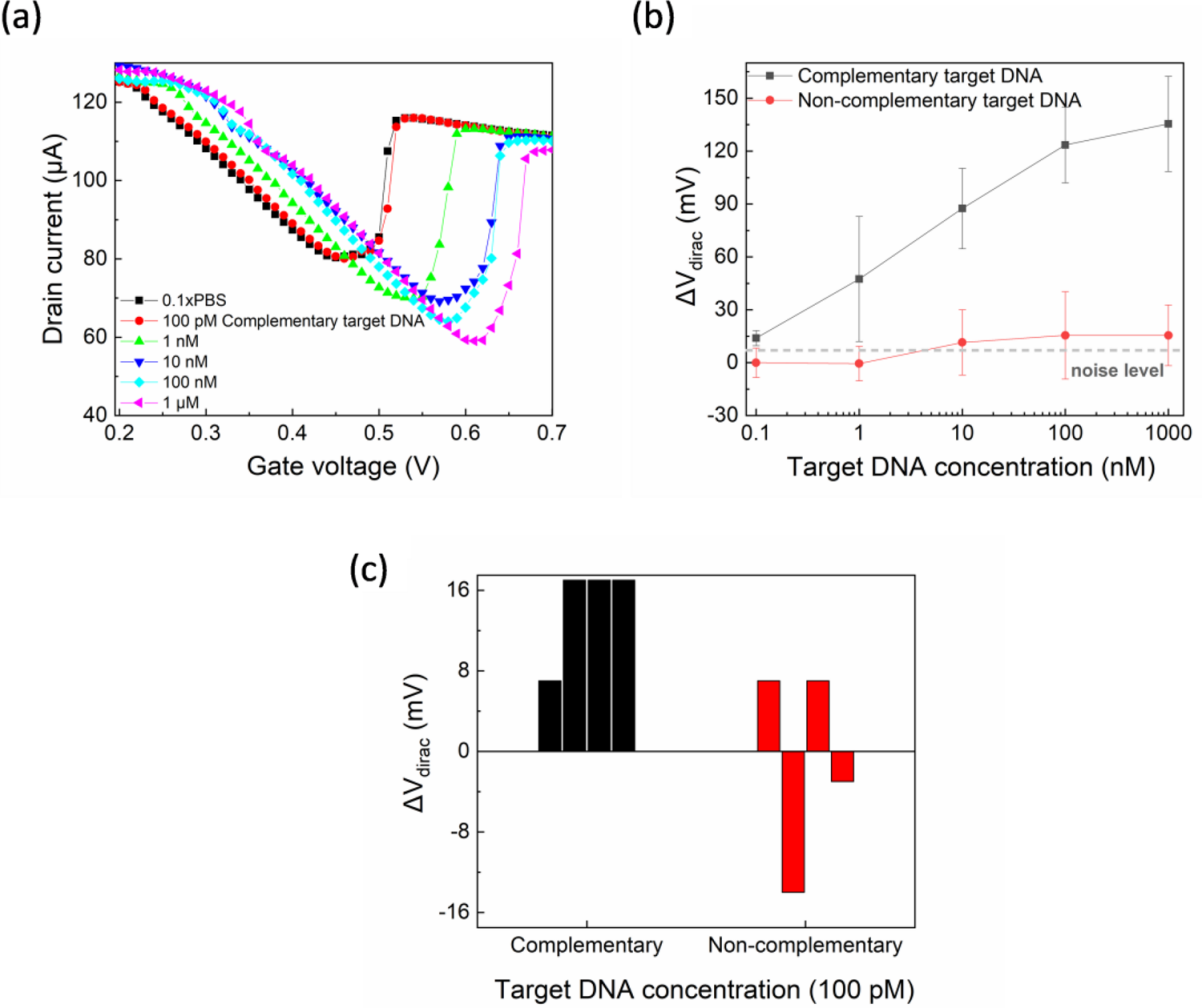
(**a**) Transfer curves for blank (0.1× PBS) and various complementary target DNA concentrations, (**b**) mean values of V_Dirac_ shifts for various complementary and non-complementary target DNA concentrations (bars represent standard deviations of 5 devices for each case), (**c**) high array yield of 8 graphene FETs; ΔV_Dirac_ data for 100 pM hybridization and control.

To verify the biosensing capability of our devices, preliminary tests were performed introducing complementary target DNA to the PNA functionalized graphene surface. Prior to the first target DNA concentration incubation, a blank control measurement was performed in 0.1× PBS. Figure 4a shows the transfer characteristics of the PNA functionalized device incubated with 0.1× PBS and successive target DNA concentrations ranging from 100 pM to 1 μM. Systematic positive shifts of the curves with increasing complementary DNA concentration are observed along with a decrease of the minimum I_ds_ value. The V_Dirac_ shift of the calculated mean values of five different devices (device-to-device standard deviation is represented by error bars) for complementary and non-complementary target DNA concentrations is summarized in Fig. 4b. The complementary target sensitivity curve is clearly distinguished from the non-complementary one even though the error bars of the former are not negligible. The flat sensitivity curve of the non-complementary DNA sequence verifies that our EGGFETs response specifically to the binding of the complementary target DNA. Dashed line in Fig. 4b represents the noise level (7.5 mV) from the blank control test. The limit of detection (LoD) is 1 nM and was achieved by considering the 3× noise level. Significantly, a lower LoD of 100 fM has been reported for drop-casted graphene related material on SiO_2_/Si chips using standard semiconductor technology^30^. The LoD of our devices is expected to be relatively high as these are preliminary results without biochemistry optimization^44^ or microfluidic integration^31,45–47^; yet the ssDNA detection at this concentration range is successful with higher sensitivity (30.1 mV/decade) than electrolyte gated FETs with CVD graphene on glass, decorated with Au nanoparticles (~ 16.3 mV/decade)^25^. Table 1 summarizes the characteristics of previously reported EGGFETs. PCB substrate is exploited only in this work.

**Table 1:** Literature survey of graphene-based electrolyte-gated FET DNA biosensor characteristics.

Reference	Substrate	Graphene	Reference electrode	LoD	Sensitivity (mV/decade)
Dong et al.^25^	Glass	CVD grown, Au NP decorated	External	> 10 pM	~ 16.3
Manoharan et al.^48^	SiO_2_/Si	Mechanical exfoliation	Integrated/Planar	> 10 nM	–
Cai et al.^30^	SiO_2_/Si	Drop-casted rGO	External	100 fM	~ 30.4
Yin et al.^49^	SiO_2_/Si	Langmuir–Blodgett method, rGO, Pt functionalized	External	2.4 nM	–
Chen et al.^34^	SiO_2_/Si	CVD grown	External	1 pM	~ 14.5
Xu et al.^50^	SiO_2_/Si	CVD grown	External	100 fM	–
Campos et al.^23^	SiO_2_/Si	CVD grown	Integrated/Planar	~ 25 aM	24
This work	PCB	Drop-casted ink	Integrated/Planar	1 nM	30.1

The potential for implementation of multiple, parallel sensor DNA microarrays was further explored and highlighted in Fig. 4c; such an implementation could further enhance the sensitivity and LoD of an EGGFET-enabled Lab-on-PCB platform, by incorporating appropriate statistical analysis algorithms. Even though device-to-device variability is demonstrated in Fig. 4b, Fig. 4c highlights that by measuring a group of sensors on the sensor array, we can eliminate the device variability and obtain a signal which is statistically significant even for the lowest tested complementary DNA concentration (100 pM). Τhe signals of four EGGFETs (black columns) incubated with the complementary DNA concentration are compared to the respective non-complementary DNA induced signals of another four devices (red columns) and they can indeed be clearly distinguished in batches. The signal response manifestation of a sensor array may achieve improved detection against the usual approach of relying on the construction of a single, robust and ultra-sensitive device^50,51^. This concept is highly compatible with our planar device layout and the inherent upscaling potential with the electronics-integration of the PCB technology.

The detection mechanism is attributed to the local gating induced by the negatively charged, hybridized ssDNA^23,31,52,53^. The electrostatic field of the target DNA dopes graphene with holes and the V_Dirac_ is shifted to more positive V_gs_. Positive shifts of the V_Dirac_ upon ssDNA hybridization has been also reported by other works with the same linker molecule (PASE)^23,31,53^, apart from Cai et al.^30^ who reported a left shift for their reduced graphene oxide (rGO) FETs. It can be speculated that the interaction between rGO and PASE depends on the size or oxygen content of the rGO sheets, which in turn affects the morphology of the probe PNA anchored on the channel surface. The spacer bases (TTT) of our PNA sequence also contribute to the PNA-DNA hybridization robustness by giving more flexibility to the bottom bases to interact with the complementary DNA. In addition, considering that the Debye length in Cai et al.^30^ was reduced compared to this work (due to the more heavily concentrated measuring electrolyte (1× PBS)), the reported left shift can be attributed to direct electron transfer from the electron-rich, aromatic nucleotide to rGO as a result of not properly tethered PNA-DNA hybridized strands and increased screening of the negatively charged ssDNA^20,25,52^.

In addition to the V_Dirac_ shift, the minimum I_ds_ also decreased with increasing target DNA concentration (Fig. 4a). It is worth noting that the minimum current did not exhibit a repeatable decreasing trend among the tested devices and the lack of suitability of this parameter for DNA hybridization detection has also been supported by previous reports^25^ as it is significantly affected by non-specific binding of charged molecules or ions. Finally, the severely asymmetric transfer curves for the n-type and p-type branches with the flattened n-type branch that Fig. 4a depicts can be explained by the Schottky barrier mechanism^54^. More specifically, PNA probes or ssDNA sequences are adsorbed on the metal contact and alter the contact-to-channel workfunctions difference leading to asymmetric conductance at the two branches^54^.

## Conclusions

In this work, we demonstrate for the first time electrolyte gated field-effect transistors (FETs) implemented on commercially fabricated printed circuit boards (PCBs). Graphene ink was drop-casted to form the transistor channel and one of the most commonly mutated oncogenes in human cancers was used as the target analyte to evaluate the device suitability for high-sensitivity biosensing applications. The electrical characteristics and the biosensing performance were assessed while the underlying mechanisms of transfer curve hysteresis and DNA detection were investigated and critically compared to the conventional silicon substrate based EGGFETs. This work proposes for the first time that Lab-on-PCB platforms could fully exploit their inherent electronics-compatibility to further improve sensor limit of detection, sensitivity and repeatability, via printing active electronic devices and implementing signal-post-processing algorithms on-chip in parallelized EGGFET arrays. Further advancement could be achieved via the optimization of the graphene functionalization protocol (e.g. optimum PNA probe to ethanolamine ratio) and microfluidic integration. Seamless integration with already demonstrated sample preparation, Lab-on-PCB components (e.g. μPCR-on-PCB device for rapid DNA amplification) provides a realistic route for detecting minimal DNA concentrations in commercially manufactured Lab-on-Chip devices^4,55^; such sensitivity levels can prove critical for the realization of high specification applications, such as cell-free nucleic acid cancer biomarker quantification. In addition, this work proves for the first time the feasibility of seamlessly integrating semiconductor devices in Lab-on-PCB platforms^56^ via inkjet-printing, paving the way for inkjet-printed, Lab-on-PCB platforms powered by printed electronic circuits of much higher complexity and processing power.

## Supplementary Information

Supplementary Information.
