# Supplementary material for: Printable graphene BioFETs for DNA quantification in Lab-on-PCB microsystems
Source: Sci Rep. 2021 May 10;11:9815. doi: 10.1038/s41598-021-89367-1 (PMC8111018; doi:10.1038/s41598-021-89367-1)
Supplement: Supplementary file 1 — Supplementary Information. [file 41598_2021_89367_MOESM1_ESM.docx]

Supporting Information

**Printable graphene BioFETs for DNA quantification in Lab-on-PCB microsystems**Sotirios Papamatthaiou*, Pedro Estrela, Despina Moschou

Centre for Biosensors, Bioelectronics and Biodevices (C3Bio) and Department of Electronic & Electrical Engineering, University of Bath, Bath, BA2 7AY, United Kingdom

* E-mail: spapamat@bath.ac.uk


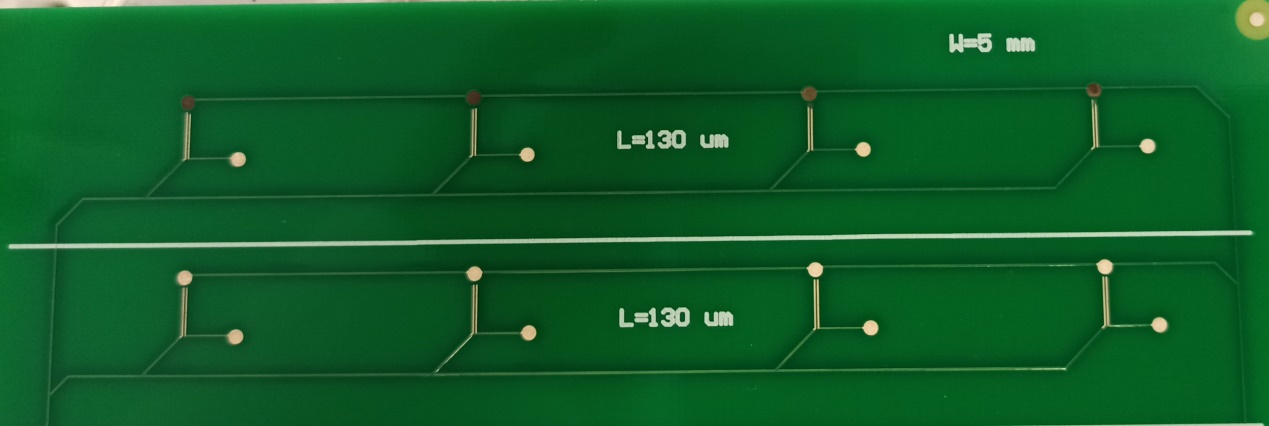


**Figure S1.** Image of non-chlorinated (bottom row) and chlorinated (top row) silver reference electrodes.





**Figure S2.** V_Dirac_ position of an electrolyte gated graphene FET determined in different concentrations of PBS for varying scan rate.
